# Total parathyroidectomy with forearm autotransplantation in secondary hyperparathyroidism patients: analysis of muscle, subcutaneous and muscle + subcutaneous method

**DOI:** 10.1186/s12893-021-01222-2

**Published:** 2021-05-01

**Authors:** Bin Zhou, Lei Zhu, Cheng Xiang, Feng Cheng, Xi Zhu, Yi Zhou, Yong Wang

**Affiliations:** 1 Department of Thyroid Surgery, The Second Affiliated Hospital of Zhejiang University College of Medicine, 88 Jiefang Road, Hangzhou, 310009 Zhejiang China; 2 Department of Thyroid and Breast Surgery, Lishui Hospital of Zhejiang University, Lishui, 323000 Hangzhou China

**Keywords:** Forearm autotransplantation, Secondary hyperparathyroidism, Muscle and subcutaneous transplantation

## Abstract

**Background:**

Forearm autotransplantation after parathyroidectomy has turned into the standard method for secondary hyperparathyroidism (SHPT) treatment in chronic kidney disease patients. Our study aimed to explore the effects of three methods including muscle, subcutaneous and muscle + subcutaneous transplant methods on SHPT.

**Methods:**

Seventy five SHPT patients were enrolled and assigned into the muscle + subcutaneous (M + S) (n = 26), muscle (M) (n = 35), and subcutaneous (S) (n = 14) groups. The operation efficacy evaluation included preoperative and postoperative biological characteristics such as parathyroid hormone (PTH), serum phosphorus, serum calcium and alkaline phosphatase (ALP). The data were recorded from pre-operation time point to 1, 2, 3, 6, 12, 18, 24 month (mo) postoperation periods. After operation, short-form health survey (SF-36) scores was made for life quality identification at 1, 2, 3, 6, 12, 24 time points. Symptoms about SHPT including bone pain, fracture, pruritus, and coronary artery calcification were followed-up based on the scale.

**Results:**

Compared with the preoperative record, all the M + S, M, and S groups showed postoperative decreased levels of PTH, serum phosphorus, serum calcium, calcium-phosphorus. In M + S group, the PTH and serum calcium level kept more steady compared with the M and S groups during a 24 mo duration observation. After this, a SF-36 score scale which represents the life quality show M + S group got more scores at 3, 6, 12, 18 and 24 mo points. At last, the incidence of SHPT associated symptoms including Bone pain, Fracture, Pruritus, and Coronary artery calcification in M + S group were decreased compared with M and S groups at 1, 3, 6, 12 and 24 mo post-operation time points.

**Conclusion:**

M + S seems to be an efficient method for medical treatment of SHPT patients in the control of PTH and serum calcium. This mixed transplant strategy improves the biochemical characterizes compared with M and S groups in SHPT patients. Furthermore, the M + S method make beneficial on clinical outcomes and life quality of patients.

## Background

Secondary hyperparathyroidism (SHPT), a common complication in end-stage renal disease patients. The hyperparathyroidism condition often persists even after successful renal transplantation with normal biochemical criterion [1]. SHPT is a symptom of increase synthesis and secretion of parathyroid hormone (PTH), and progressive parathyroid gland hyper function [2].

Fundamentally, the etiology of SHPT is known as long-term parathyroid hyperplasia, which results in the formation of functionally independent adenoma [3]. SHPT in patients can lead to severe bone disease, vascular and interstitial calcifications, as well as undesired mortality and morbidity of cardiovascular disease [4–7]. Usually, parathyroidectomy (PTX) results in a obviously reduction of serum PTH, SHPT improve and reduce the risk of bone lesion and cardiovascular diseases [8, 9]. Substantially, low PTH levels that are usually found postoperatively and may predispose a patient to adynamic bone disease and reduce the patient’s life quality due to the clinical symptoms including fractures, vascular calcifications and bone pain [10].

For the reason of the frequent occurrence and recurrence of SHPT, the patients’ quality of life is seriously affected by symptoms related to intolerable parathyroid hormone after surgery [11]. Therefore, many efforts have been made to search better solutions for SHPT. Commonly recognized techniques for parathyroidectomy consist of subtotal PTX and total PTX with forearm autotransplantation (FAT). The former removes all parathyroid tissue and leaves a portion of a normal parathyroid gland in the neck, and the latter can be helpful in removing all parathyroid tissue and also implanting small pieces of tissue at a distance in well-vascularized muscular structures [12, 13].

Parathyroidectomy accompanied with autotransplantation has been already reported with relative different results. Recently, the forearm transplantation is known as the first choice of parathyroid autotransplantation method in condition of renal hyperparathyroidism [14]. Forearm autotransplantation allows the measurement of differential parathyroid hormone levels at the antecubital veins of the arm [15, 16]. Forearm autotransplantation is usually performed in the position of muscular or subcutaneous tissue. These two methods have been applied for many years [17, 18]. Researches of evaluating forearm parathyroid autografts site shown comparable results regarding short-term graft function, in the muscular or subcutaneous tissue method [19]. Here, the subcutaneous and muscle autotransplantation methods were combined together. The aim of the study was to evaluate whether combination of these two methods could be more benefit to SHPT patients’ life quality and biochemical profiles than separate ways.

## Methods

### Ethics statement and patients enrollment

The study was approved by the Ethics Committee of Li Shui Central Hospital and written informed consents were obtained from all patients. From February 2016 to February 2018, a total of 75 uremic patients suffered from SHPT were diagnosed and received total parathyroidectomy with forearm autotransplantation in the Li Shui Central Hospital. All subjects accepted autotransplantation method were assigned into muscle + subcutaneous (M + S, n = 26), muscle (M, n = 35) and subcutaneous (S, n = 14) groups. During the 2 years period, patients receive M + S method in the first 8 month, receive M method in the median 8 month and receive S method in the last 8 month.

**Fig. 1:**
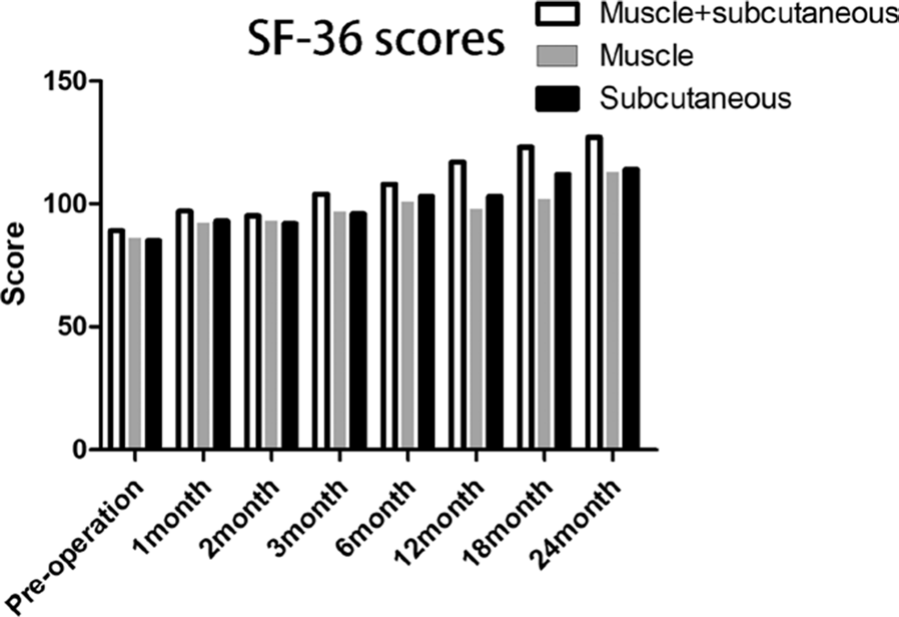
SF-36 scores in M + S, M and S groups

Inclusion criteria was as follows: (1) high levels of PTH (> 800 pg/mL) andcan not be controlled with drug treatment for 3 mo; (2) persistent hypercalcemia despite interruption of calcium and calcitriol, a calcium-phosphorus product > 70 mg^2^/dL^2^; (3) clinical manifestations presented happened that include bone deformities, pathological fractures,, disabling arthritis, intractable pruritus. Exclusion criteria included (1) previous hospitalization within 1 mo; (2) active inflammation condition; (3) malignancy tumor; (4) steroids or immunosuppressive agents applied; (5) congenital, genetic, autoimmune, or cardiovascular diseases. Informed consents were obtained from participants. The patients receive history-taking, clinical evaluation and laboratory parameters test (blood count, serum calcium test, phosphorus, PTH, magnesium, and albumin levels) in preoperative and postoperative, respectively (Fig. 1).

### Preparations and surgical procedure

**Fig. 2:**
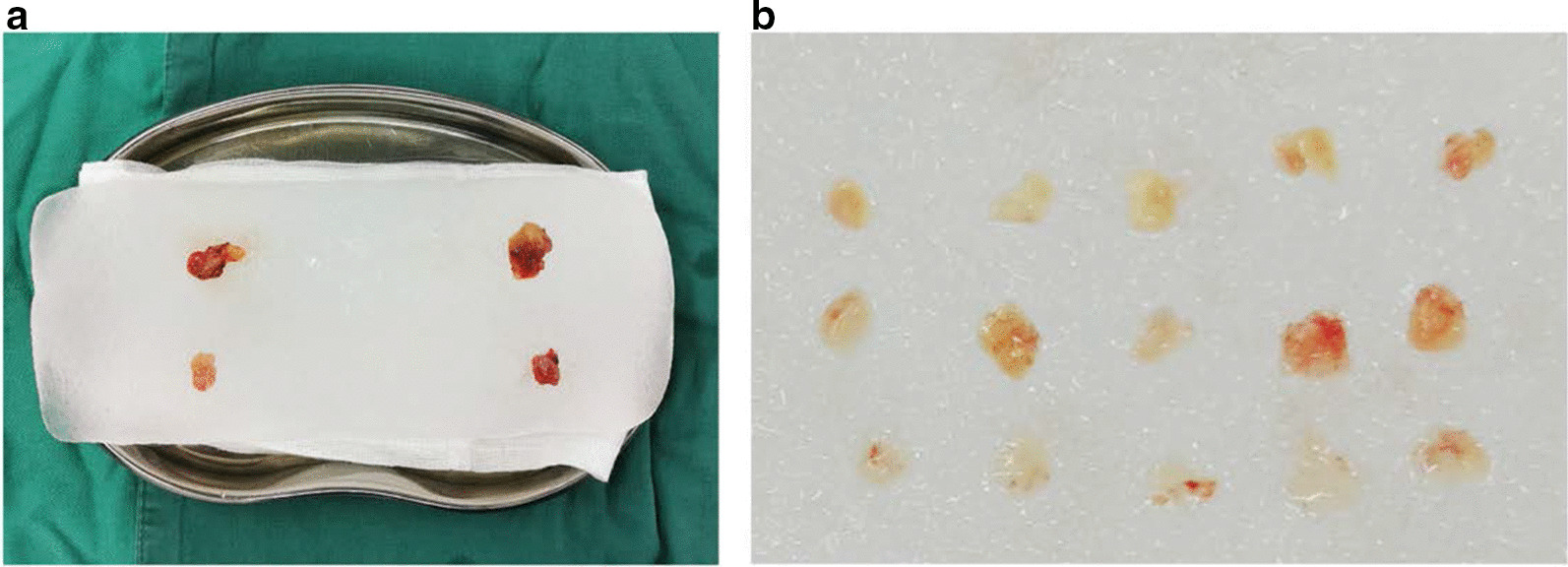
Parathyroid pieces preparation. **a** Four Parathyroid glands were confirmed and dissected **b** One parathyroid gland was cut into 15 1 × 1 mm pieces

1 week before surgery, all patients were hospitalized before surgery. They take calcium tablets (500 mg three times/day) and alfacalcidol (1 μg/day). Patients received dialysis without heparin 6 to 12 h before operation. Blood samples were collected and serum calcium, serum phosphate, PTH, and ALP. B ultrasound were performed to localize the hyperplastic gland before operation. During operation, hyperplastic parathyroid gland frozen section biopsy was performed after excision to confirm the parathyroid tissue. The hyperplastic gland was then transfered within a sterile cup containing 4 °C saline (Fig. 2a, b). The opposite site of the fistula forearm was selected for implant site, and prepped and exposed at the initial of the case.

At the point that all the hyperplastic glands were dissected, the level of serum intraoperative PTH decreased appropriately with rapid serum PTH test during surgery. The hyperplastic gland for implantation was prepared for selection. Usually, the smallest nodele gland was preferable. Glands transplant should be avoided when the nodular glands showed a monoclonal pattern in clonal analysis, which was more aggressive and tumor like that more likely led to graft dependent hyperparathyroidism.

**Fig. 3:**
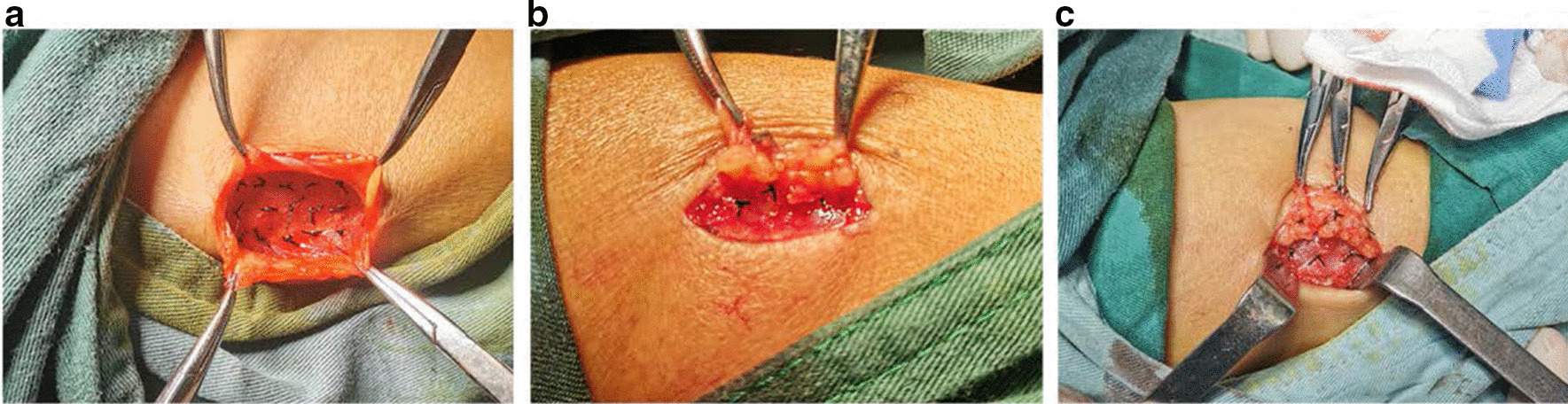
Parathyroid transplant procedure. **a** M group **b** S group **c** M + S group

In our study, three groups of M + S, M, and S were set to conduct transplant. The dissected parathyroid tissue was cut into a 15 portion of 1 × 1 × 1 mm volume pieces. The 1 cm incision was made in a vertical row on aspect of the forearm for parathyroid transplant, and the managed parathyroid tissue was then transplanted into the muscle pockets or the subcutaneous pockets in the incision. In M + S group, 9 pieces parathyroid tissue were transplanted into 3 different subcutaneous pockets, and another 6 pieces were transplanted into 3 muscle pockets. In M group, 15 pieces parathyroid tissue were transplanted into 15 muscle pockets. In S group, 15 pieces were individually transplanted into 3 different subcutaneous pockets. After these, the pockets were sutured with non-absorbable suture after hemostasis in prepare for parathyroid tissue positioning in the second surgery (Fig. 3a–c).

### Postoperative follow-up

After surgery, patients were maintained with alfacalcidol (1 μg daily), calcium tablets in prevention of low calcium (400 mg; two tabs three times/day, in a combination containing magnesium hydroxide, zinc, and vitamin D). After perioperative period, patients continued to take calcium tablets (500 mg, two tabs, three times daily) and were guided weekly for measurements of calcium levels. After PTX, we assessed serum calcium, phosphate and PTH levels on post-operation moment and 1, 2, 3, 6, 12, 18, 24 mo points. Symptoms related to hyperthyroid such as Bone pain, Fracture, Pruritus and Coronary artery calcification were observed before operation and 1, 3, 6, 24 mo after surgery.

### Statistical analysis

We performed all statistical analyses using SPSS version 17.0 (SPSS Inc, Chicago, IL) software package. Continuous variables were expressed as median. Categorical and ordinal variables were expressed as numbers. T test was adapted for the comparison between 2 groups, and repeated measurement analysis of variance (ANOVA) for comparison between 2 groups at different time points. *P* < 0.05 was considered as statistical significance.

## Results

### Baseline characteristics among M + S, M, and S groups

As presented in Table.1, a total of 75 patients were included, 26 patients received M + S surgery, 35 patients were subjected to M surgery and 14 patients were performed with S surgery. Difference of sex and age among 3 groups were not statistically significant (*P* > 0.05). Dialysis methods included Hemodialysis and Peritoneal. The dialysis method ratio, duration of dialysis before surgery and follow-up duration were not statistically significant among the M + S, M and S groups (*P* > 0.05).

**Table 1:** Preoperative demographic and clinical data of the study participants (n = 75)

Characteristics	Muscle + subcutaneous	Muscle	Subcutaneous	*P*-value
Sex				
Male	11	15	9	0.059
Female	15	20	5	
Total	26	35	14	
Age (year)	48.9 ± 9.7	48.6 ± 10.7	50.0 ± 6.7	0.84
Dialysis method				
Hemodialysis	15	16	7	0.651
Peritoneal	11	19	7	
Duration of dialysis (year)	5.9 ± 1.6	6.4 ± 2.1	6.0 ± 2.2	0.68
Follow up (Month)	27.6 ± 3.7	28.2 ± 5.2	26.3 ± 3.3	0.92

### Biochemical profiles among M + S, M and S group

In table.2, the biochemical profiles before and after parathyroidectomy were recorded. Calcium level decreased after surgery in M + S group (2.32 ± 0.37 mmol/L vs 2.15 ± 0.26 mmol/L), M group (2.36 ± 0.27 mmol/L vs 2.23 ± 0.34 mmol/L), and S group (2.27 ± 0.47 mmol/L vs 2.14 ± 0.39 mmol/L). However, the calcium level was not significantly different among M + S, M and S groups before (*P* = 0.64) and after surgery (*P* = 0.72).

**Table 2:** Biochemical profiles of the patients

Characteristics	Muscle + subcutaneous	Muscle	Subcutaneous	*P*-value
Calcium (mmol/L) Normal range: 2.05–2.48				
Pre-operation	2.32 ± 0.37	2.36 ± 0.27	2.27 ± 0.47	0.64
Post-operation	2.15 ± 0.26	2.23 ± 0.34	2.14 ± 0.39	0.72
Phosphate (mmol/L) Normal range: 0.80–1.33				
Pre-operation	2.32 ± 0.26	2.33 ± 0.28	2.38 ± 0.45	0.54
Post-operation	1.61 ± 0.25	1.71 ± 0.17	1.64 ± 0.15	0.43
PTH (pmol/L) Normal range: 15.00–65.00				
Pre-operation	1336.70	1546.60	1441.85	0.48
Post-operation	19.800	43.300	16.600	0.04
ALP (U/L) Normal range: 30.00–62.00				
Pre-operation	325.02 ± 57.37	298.75 ± 46.28	287.25 ± 65.23	0.65
Post-operation	145.63 ± 32.28	165.65 ± 43.28	165.48 ± 38.35	0.13

The PTH value was presented as median, and others parameters were presented as means ± standard deviation

Before operation, serum phosphate was significantly higher in M + S (2.32 ± 0.26 mmol/L), M (2.33 ± 0.28 mmol/L) and S (2.38 ± 0.45 mmol/L) groups compared with normal range (0.8–1.33 mmol/L). After surgery, the serum phosphate slightly decreased in M + S (1.61 ± 0.25), M (1.71 ± 0.17) and S (1.64 ± 0.15) groups.

PTH levels were 1492.64 ± 387.27, 1553.08 ± 478.28 and 1488.41 ± 376.39 pmol/L in M + S, M and S groups, respectively (*P* = 0.36). After all the secondary hyperparathyroidism patients that followed the indication in guidelines received surgery, PTH decrease to 30.06 ± 12.65, 112.46 ± 37.27 and 50.54 ± 15.27 pmol/L in M + S, M and S group (*P* = 0.01).

In addition, ALP activation was controlled after M + S, M or S surgery and the treatment effect seemed similar among three groups.

### Long term profiles after parathyroid surgery

Table.3 presented the postoperative levels of PTH (pmol/L), Calcium (mmol/L), Phosphate (mmol/L) at different times in M + S, M and S groups after surgery.

**Table 3:** Long term profiles after parathyroid gland operation

Characteristics	1 month	2 month	3 month	6 month	12 month	18 month	24 month
PTH (pmol/L)							
Muscle + subcutaneous	21.000	51.100	30.500	30.700	20.800	30.200	50.600
Muscle	12.750	20.400	36.800	42.350	37.000	40.600	123.100
Subcutaneous	20.400	32.900	61.800	47.250	49.700	55.700	65.2000
Calcium (mmol/L)							
Muscle + subcutaneous	1.95 ± 0.24	1.98 ± 0.18	2.15 ± 0.32	2.07 ± 0.23	2.13 ± 0.32	2.23 ± 0.23	2.18 ± 0.42
Muscle	2.02 ± 0.23	2.27 ± 0.34	2.26 ± 0.46	2.18 ± 0.28	2.05 ± 0.21	2.28 ± 0.32	2.16 ± 0.23
Subcutaneous	2.13 ± 0.23	2.16 ± 0.18	2.26 ± 0.32	2.23 ± 0.28	2.17 ± 0.34	2.03 ± 0.23	2.13 ± 0.39
Phosphate (mmol/L)							
Muscle + subcutaneous	1.26 ± 0.11	1.33 ± 0.18	1.41 ± 0.23	1.29 ± 0.16	1.65 ± 0.17	1.57 ± 0.21	1.38 ± 0.12
Muscle	1.43 ± 0.16	1.28 ± 0.21	1.35 ± 0.18	1.38 ± 0.21	1.38 ± 0.23	1.53 ± 0.18	1.43 ± 0.16
Subcutaneous	1.41 ± 0.21	1.23 ± 0.22	1.34 ± 0.23	1.23 ± 0.14	1.43 ± 0.24	1.45 ± 0.26	1.34 ± 0.21

The PTH value was presented as median, and others parameters were presented as means ± standard deviation

In M + S group, the PTH kept steady during the 24 mo period. There was a gradual increase in PTH over time in M and S group. Changes of serum Calcium and Phosphate level were not observed among 3 groups. None of the patients required a second operation because of recurrence.

### Postoperative symptoms and signs among M + S, M and S groups

The signs and symptoms of each group were summarized in Table.4. Compared with preoperative values, the bone pain ratio in M + S group decreased significantly compared with M and S groups at 3, 6, 12 mo points. Compared with the M group, pruritus ratio decreased significantly in both M + S and S groups at each time point. The differences of Fractures and Coronary artery calcification were not statistically significant at all points among M + S, M and S groups.

**Table 4:** Operative symptoms and signs

Symptoms and signs	Operation methods	Pre-operation	1 month	3 month	6 month	12 month	24 month
Bone pain	Muscle + subcutaneous	25	18	14	10	8	6
	Muscle	33	32	27	22	16	10
	Subcutaneous	12	10	10	6	4	3
Fracture	Muscle + subcutaneous	8	6	6	3	2	0
	Muscle	5	4	2	2	2	0
	Subcutaneous	3	2	2	1	1	0
Pruritus	Muscle + subcutaneous	21	16	12	9	6	3
	Muscle	30	27	22	17	14	12
	Subcutaneous	9	7	4	2	2	1
Coronary artery calcification	Muscle + subcutaneous	9	3	2	0	0	0
	Muscle	12	6	2	1	1	0
	Subcutaneous	4	3	3	3	2	0

### Comparison of SF-36 scores among the M + S, M, and S groups

SF-36 scores in M + S, M and S groups were shown in Fig. 1. Compared with preoperative values, the SF-36 scores in 3 groups increased significantly at different time periods after the operation (all *P* < 0.05). In M + S group, the SF-36 scores were statistically higher than both M and S group at 1, 2, 3, 6, 12, 24 mo points (all *P* < 0.05).

## Discussion

SHPT is the common problem in the treatment of chronic kidney disease patients. Although hyperparathyroidism can be medically or surgically controlled, but severe hyperparathyroidism may increase the medical control difficulty. In 1967, Ogg reported that PTX was proved to be an effective treatment for SHPT [20]. PTX with autotransplantation led to improvement of chronic kidney disease-mineral and bone disease, so it may be applied for patients with SHPT that were resistant to vitamin D analogues and calcium treatment. PTX treatment can improve the short and long term outcomes of patient with severe SHPT [21].

Usually, forearm autotransplantation included two methods, namely, muscle and subcutaneous methods. In previous study, muscular or subcutaneous forearm parathyroid autografts shown comparable results in short-term graft function observation [19, 22]. In our study, intramuscular and subcutaneous methods were favored and reached relative satisfactory result. Subcutaneous autotransplantation was more favorable for the reason of simple procedures in second operations. In intramuscular autograftectomy group, high incidence of repeated autograftectomy were found comparison with subcutaneous autografts [23–25]. Chou et al. reported that higher incidence of autograftectomy was found in intramuscular autotransplantation (9 of 75, 12% vs 1 of 110, 0.9%) compared with subcutaneous autotransplantation has [26]. Another study indicated that intramuscular parathyroid autotransplantation havea higher incidence of autograftectomy and secondary autograftectomy (4 of 65, 6.4% vs 1 of 823, 0.1%) compared with subcutaneous parathyroid autotransplantation method [27]. Although the intramuscular group appeared to have no outcome favorable, no uniform operative technique has been accepted. For example, polyethyleneglycol-polyalanine-co-phenylalanine based thermoreversible gel was a convenience carrier material for intramuscular parathyroid autotransplantation [28]. The fact indicated that improved autotransplantation method needed to be found besides subcutaneous parathyroid implant.

In our study, we performed the intramuscular and subcutaneous methods together. The autotransplantation technique of parathyroid has been widely and historically described and practiced [29]. Generally, before transplantation, we performed the frozen section biopsy at the time that each hyperplastic parathyroid gland was removed to confirm the parathyroid tissue. After hyperplastic glands are removed, usually the intraoperative serum PTH would fall appropriately, then one of the hyperplastic gland is selectedfor further implantation. At most time, the smallest nodular gland is preferable. 1 cm vertical row incision on the forearm is made on the volar aspect, below the incision, a subcutaneous pocket is created. Under the help of microvascular instruments, fat is isolated from the transplanted gland, then the gland was cut into multiple 1 × 2 mm pieces. The illustration describ the incisions and transplant site of and subcutaneous pocket for the parathyroid tissue pieces. After hemostasis, each incision is ligtured with a subcuticular absorbable suture. In our center, we cut the parathyroid into a more tiny 1 × 1 mm pieces to improve the vascularization and gland survival. Although the dissection and transplant procedure would consume more time, Yu-Chen Hsu reported the operating times for intramuscular and subcutaneous were 79 and 37 min, respectively [27]. We found a half more hour was needed when more parathyroid pieces and transplant pockets were prepared. After operation, we speculated that the survival of parathyroid tissue in combined intramuscular and subcutaneous groups would make physiological requirement of PTH more steady compared with intramuscular and subcutaneous transplant methods alone.

Combination of PTX and FAT was a safe, feasible and effective surgical option for SHPT patients [13]. Under the help of intraoperative neuromonitoring, severe recurrent laryngeal nerve injury could be avoided for the reason that the surgical strategy would be reconsidered when loss of signal happened [30]. In this study, no death occurred among patients suffered from SHPT during the perioperative period of intramuscular or subcutaneous procedures. The results of SF-36 questionnaire indicated that the quality of life in patients accepted combined surgery significantly increased compared with individual intramuscular or subcutaneous transplantation at 1, 2, 3, 6, 12, 24 month time points. From the results, we can say that combination of intramuscular and subcutaneous transplant procedures was effective in promoting the quality of life of patients with SHPT.

Furthermore, the levels of serum calcium, phosphorus, ALP, and PTH improved after surgery, while no difference appeared among three groups. Serum calcium level dropped significantly after surgery as the result of sudden PTH decrease. After surgery, intravenous calcium supplement was recommended for patients performed parathyroid surgery in case of severe hypocalcemia symptoms such as cramps and numbness. As reported, the vitamin D supplements calcium and can prevent the postoperative hypocalcemia condition and increase the safe and early stage discharge [31]. Based on this fact, we speculated that combining intramuscular with subcutaneous transplant could improve the long-term prognosis of SHPT patient without the immediate effect.

It should be acknowledged that this study has some limitations. The sampling method of subjects in this study might cause potential impact on the results and lead to potential bias. The insufficient sample size is unfavorable for the determination of most appropriate approach for SHPT. In addition, extended follow-up may be necessary to demonstrate treatment benefit. Therefore, further studies with larger sample size and longer follow-up duration were needed to support the findings.

## Conclusions

The location of forearm parathyroid autotransplantation are the face that affects the autograft result. The present study demonstrates that intramuscular combines with subcutaneous transplant seems to be an efficient treatment option for SHPT. Subcutaneous group shows no advantage towards intramuscular in both short-term and long-term observation, which is contradict to previous studies. This may result from the more fragmented management before transplantation improving the intramuscular transplant quality.

## Data Availability

The datasets generated and/or analyzed during the current study are not publicly available due to restrictions by the local Institutional Review Board, because further study containing related data in this study is not ended yet. But the data are available from the corresponding author upon reasonable request and with permission from the local Institutional Review Board.

## Abbreviations

SHPT: Secondary hyperparathyroidism
ALP: Alkaline phosphatase
PTH: Parathyroid hormone
PTX: Parathyroidectomy
ABD: Adynamic bone disease
FAT: Forearm autotransplantation
mo: Month
